# Soy Isoflavones Inhibit Both GPIb-IX Signaling and αIIbβ3 Outside-In Signaling via 14-3-3ζ in Platelet

**DOI:** 10.3390/molecules26164911

**Published:** 2021-08-13

**Authors:** Ming Liu, Gan Wang, Runjia Xu, Chuanbin Shen, Heyu Ni, Ren Lai

**Affiliations:** 1 Department of Molecular and Cell Biology, School of Life Sciences, University of Science and Technology of China, Hefei 230027, China; qhdming@mail.ustc.edu.cn; 2 Key Laboratory of Bioactive Peptides, Yunnan Province/Key Laboratory of Animal Models and Human Disease Mechanisms, Kunming Institute of Zoology, Chinese Academy of Sciences, Kunming 650032, China; wang.gan@outlook.com (G.W.); xurunjia1991@163.com (R.X.); 3 Department of Laboratory Medicine and Pathobiology, University of Toronto, Toronto, ON M5S 1A1, Canada; shenchuanbin@126.com (C.S.); Heyu.Ni@unityhealth.to (H.N.); 4 Department of Laboratory Medicine, LKSKI-Keenan Research Centre for Biomedical Science, St. Michael’s Hospital, Toronto, ON M5B 1W8, Canada; 5 Toronto Platelet Immunobiology Group, Toronto, ON M5B 1W8, Canada; 6 Department of Physiology, University of Toronto, Toronto, ON M5S 1A1, Canada; 7 Canadian Blood Services Centre for Innovation, Toronto, ON M5G 2M1, Canada; 8 Department of Medicine, University of Toronto, Toronto, ON M5S 1A1, Canada

**Keywords:** soy, isoflavone, daidzein, 14-3-3ζ, platelet, GPIb-IX, αIIbβ3

## Abstract

Soy diet is thought to help prevent cardiovascular diseases in humans. Isoflavone, which is abundant in soybean and other legumes, has been reported to possess antiplatelet activity and potential antithrombotic effect. Our study aims to elucidate the potential target of soy isoflavone in platelet. The anti-thrombosis formation effect of genistein and daidzein was evaluated in ex vivo perfusion chamber model under low (300 s^−1^) and high (1800 s^−1^) shear forces. The effect of genistein and daidzein on platelet aggregation and spreading was evaluated with platelets from both wildtype and *GPIbα* deficient mice. The interaction of these soy isoflavone with 14-3-3ζ was detected by surface plasmon resonance (SPR) and co-immunoprecipitation, and the effect of αIIbβ3-mediated outside-in signaling transduction was evaluated by western blot. We found both genistein and daidzein showed inhibitory effect on thrombosis formation in perfusion chamber, especially under high shear force (1800 s^−1^). These soy isoflavone interact with 14-3-3ζ and inhibited both GPIb-IX and αIIbβ3-mediated platelet aggregation, integrin-mediated platelet spreading and outside-in signaling transduction. Our findings indicate that 14-3-3ζ is a novel target of genistein and daidzein. 14-3-3ζ, an adaptor protein that regulates both GPIb-IX and αIIbβ3-mediated platelet activation is involved in soy isoflavone mediated platelet inhibition.

## 1. Introduction

A systematic analysis for the Global Burden of Disease Study revealed that cardiovascular disease (CVD) has the highest mortality among all the non-communicable diseases [1]. Thrombus is responsible for high-mortality CVD such as myocardial infarction and stroke, and platelets play important roles in the pathogenesis of these diseases [2,3,4]. The inhibition of platelet function has been used for a long time to prevent and treat CVD [5]. However, anti-platelet drugs such as aspirin and clopidogrel increase bleeding risk and may precipitate a hemorrhagic stroke and therapeutic resistance [6], which greatly limits the use of these drugs.

The well-known correlation between diet and health provides strong evidence that functional foods may maintain or improve health and prevent CVD [6,7,8]. Soybeans and related food products have been an integral part of regular diets all over the world, especially in Asian countries [7,9]. Epidemiological studies suggested that soy consumption is associated with a lower incidence of CVD [10,11,12]. Therefore, based on clinical trials and epidemiological data, the US Food and Drugs Administration also approved a health claim for soy [13]. Natural isoflavones mostly exist in bean family plants, and they are particularly abundant in soybeans. Dietary soy isoflavones rather than soy proteins exert antiplatelet functions in agonist-induced platelet activation [14,15].

Early studies have shown that isoflavones might exert anti-platelet function through cAMP regulation [16], tyrosine kinase [17], calcium messenger [18], and TxA_2_ pathway inhibition [19]. Moreover, genistein and daidzein can prolong the time that thrombotic vessel occlusion was produced in the femoral arteries of mice using the photochemical thrombosis model [20], and they have a good protective effect on the death or paralysis of mice caused by the pulmonary embolism model induced by the injection of collagen and epinephrine in the tail vein [21]. Although multiple factors may be involved in isoflavone-mediated platelet inhibition, the direct target of soy isoflavone in platelet needs further investigation. Our previous study revealed 14-3-3ζ is important in platelet integrin outside-in signaling and is a potential target for antithrombotic therapy without causing significant bleeding. An isoflavone which identified from a library (LR-NP1792; Mendeley Data doi:10. 17632/2kbjft8jd3.1) targets 14-3-3ζ [22]. In the present study, we reported that soy isoflavones (genistein and daidzein) directly bind to 14-3-3ζ protein and inhibit both GPIb-IX and αIIbβ3 mediated platelet activation.

## 2. Results and Discussion

### 2.1. Genistein and Daidzein Inhibit Platelet Aggregation and Thrombi Formation in an Ex Vivo Thrombosis Model

**Figure 1:**
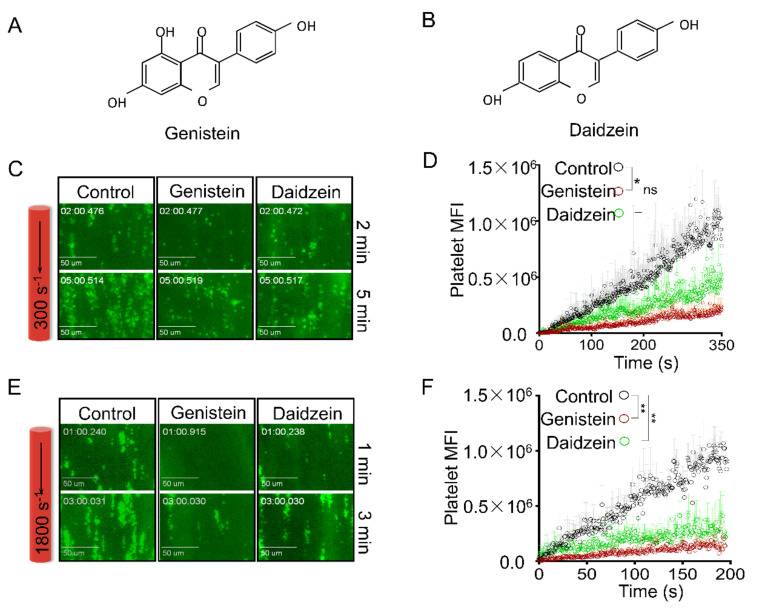
Genistein and daidzein inhibited thrombus formation in perfusion chamber. The structural formula of genistein (**A**) and daidzein (**B**). The representative images (**C**) and quantification (**D**) of platelet adhesion and aggregation at 300 s^−1^ shear rate for 5 min, the concentration of genistein and daidzein both are 100 μM. The platelets were incubated with isoflavone compounds and their solvent (DMSO) for 5 min at 37 °C. The representative images (**E**) and quantification (**F**) of platelet adhesion and aggregation at 1800 s^−1^ for 3 min. The circle represent platelet mean fluorescence intensity (MFI) and the shaded regions represent SEM of three independent experiments. Statistical analysis was performed using nonparametric test with a Dunn’s multiple comparison test. Significance was defined as * *p* < 0.05, ** *p* < 0.01. Green dots: fluorescent platelets. Bar = 50 μm.

Many isoflavones and their analogues have been demonstrated to inhibit platelet activation [17,19]. However, the effect of soy isoflavones on real-time thrombus formation under different shear stresses has seldom been studied. Here we found that in heparinized mouse blood, platelet-mediated thrombi formed more rapidly under a higher shear rate (1800 s^−1^) in collagen-coated perfusion chamber. Both genistein (Figure 1A, CAS registry numbers are 446-72-0) and daidzein (Figure 1B, CAS registry numbers are 486-66-8) inhibited thrombi formation under the shear rate of 300 s^−1^ (Figure 1C,D) and 1800 s^−1^ (Figure 1E,F), while genistein showed higher potency. However, these isoflavones were preferred to inhibit the platelet aggregation and thrombosis at higher shear rate (1800 s^−1^), suggesting these compounds may have an inhibitory effect on platelet mechanosensor GPIb-IX mediated signaling transduction [23,24].

### 2.2. Genistein and Daidzein Inhibit GPIb-IX-Mediated Platelet Aggregation

**Figure 2:**
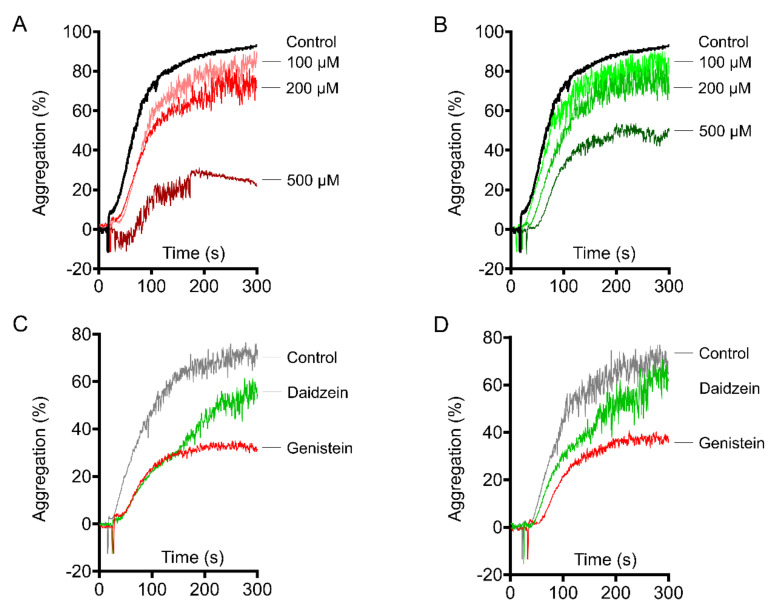
The platelets were incubated with isoflavone compounds and their solvent (DMSO) for 5 min at 37 °C. Genistein and daidzein inhibited GPIb-mediated platelet agglutination. Genistein (**A**) and daidzein (**B**) concentration-dependently inhibited 1.2 mg/mL ristocetin induced platelet aggregation in mice PRP. Genistein and daidzein inhibited ristocetin-induced platelet agglutination in the presence of 1 mM integrilin (**C**) and 5 mM EDTA (**D**).

It has been well characterized that von Willebrand factor binds to collagen and promotes thrombus formation under high shear force via GPIb-IX signaling [25,26,27]. We found that genistein and daidzein significantly reduced platelet-mediated thrombi formation under high shear force (Figure 1D). Therefore, we investigated whether these compounds affect GPIb-IX-mediated platelet aggregation. Genistein (Figure 2A) and daidzein (Figure 2B) concentration-dependently inhibited ristocetin-induced platelet aggregation. However, Ristocetin-induced platelet aggregation involves GPIb-IX-mediated platelet agglutination and subsequent integrin αIIbβ3-mediated platelet aggregation [28]. To exclude integrin-mediated platelet aggregation, we examined the effect of the soy isoflavones on ristocetin-induced platelet agglutination in the presence of eptifibatide (Figure 2C) or EDTA (Figure 2D), which abrogates integrin-dependent ligand binding [29]. Both genistein and daidzein inhibited ristocetin-induced platelet agglutination in the presence of 1 mM eptifibatide or 5 mM EDTA, suggesting these isoflavones inhibit GPIb-IX signaling independent of integrin.

### 2.3. Genistein and Daidzein Inhibit αIIbβ3-Mediated Platelet Aggregation

**Figure 3:**
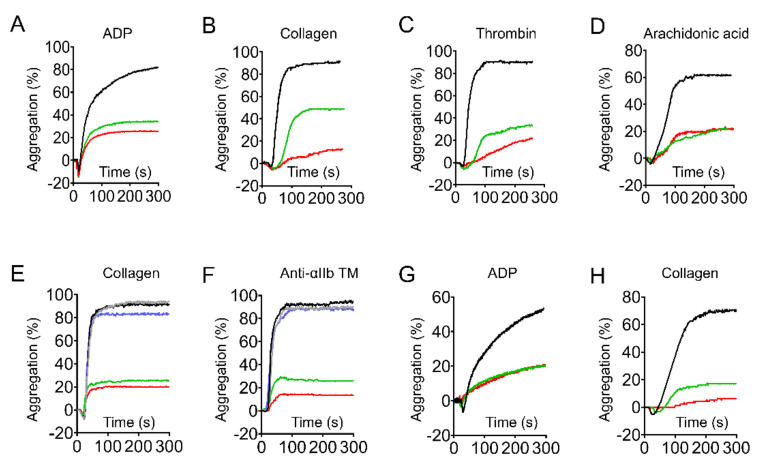
Genistein and daidzein inhibited integrin-mediated platelet aggregation. The platelets were incubated with isoflavone compounds and their solvent (DMSO) for 5 min at 37 °C. Genistein and daidzein at 200 μM inhibited 5 μM ADP-induced platelet aggregation in PRP (**A**). Genistein and daidzein at 200 μM inhibited 2 μg/mL collagen (**B**), 0.04 U/mL thrombin (**C**), and 3 μM arachidonic acid (**D**)-induced platelet aggregation in washed platelets. Effect of genistein, daidzein, SQ29548 (3 μM), and apyrase (0.5 U/mL) on 50 μg/mL collagen (**E**) or 1 μg/mL anti-αIIbTM (**F**)-induced platelet aggregation in the presence of 100 μg/mL fibrinogen. Genistein and daidzein inhibited 5 μM ADP (**G**) and 2 μg/mL collagen (**H**)-induced platelet aggregation in *GPIbα*-deficent mice. Black curve: control. Red curve: genistein treated. Green curve: daidzein treated. Blue curve: SQ29548 treated. Grey curve: apyrase treated.

Platelets from wild type and *GPIbα* deficient mice were used to investigate the effect of genistein and daidzein on αIIbβ3-mediated platelet aggregation. We found that not only genistein and daidzein inhibited ADP (Figure 3A), collagen (Figure 3B), thrombin (Figure 3C), and arachidonic acid (Figure 3D) induced platelet aggregation but also high concentration of collagen (Figure 3E) and integrin αIIbβ3 activator anti-αIIb TM (Figure 3F) induced platelet aggregation, suggesting these soy isoflavones might inhibit αIIbβ3 outside-in signaling [22,30,31]. The inhibitory effect of genistein and daidzein on platelet aggregation was not through GPIb-IX signaling, since both of these compounds inhibited ADP (Figure 3G) and collagen (Figure 3H) induced platelet aggregation in *GPIbα* deficit platelets.

### 2.4. Genistein and Daidzein Inhibit Platelet Spreading on Immobilized Fibrinogen

**Figure 4:**
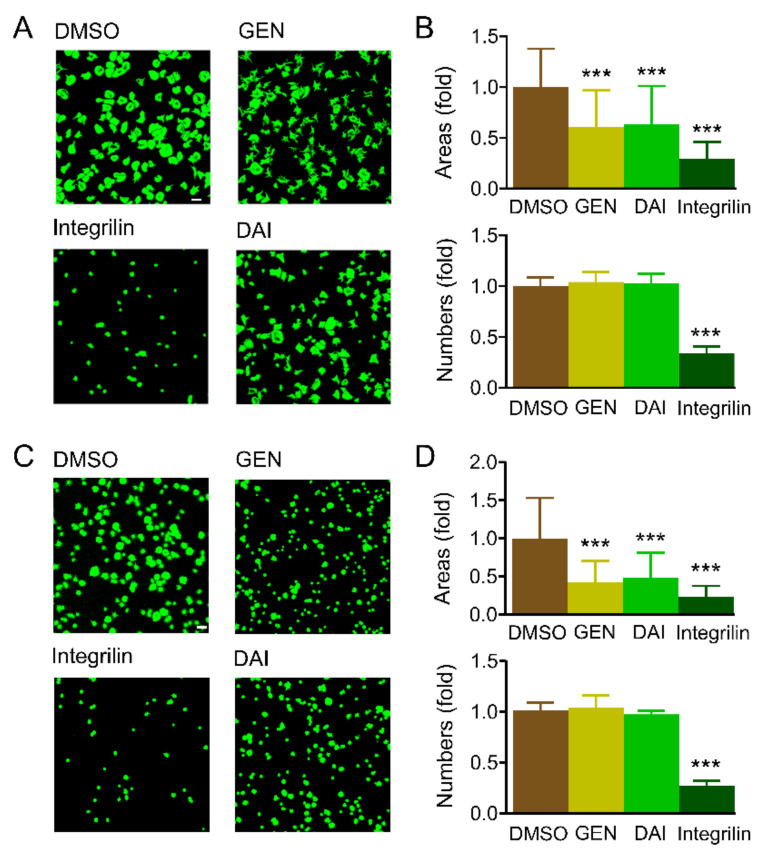
Genistein and daidzein inhibited integrin-mediated platelet spreading. (**A**,**B**) Confocal microscopy of platelet adhesion and spreading on immobilized fibrinogen. Washed wild type platelets pre-added with 0.1 % DMSO, 100 μM genistein (GEN) and daidzein (DAI), 3 μM integrilin were allowed to spread on immobilized fibrinogen for 40 min at 37 °C, then stained with FITC-conjugated phalloidin. Scale bar = 10 μm. (**C**,**D**) Confocal microscopy of *GPIbα*-deficient platelet adhesion and spreading on immobilized fibrinogen. Scale bar = 20 μm. Platelet adhesion numbers were calculated with 3 random vision fields, and spreading areas were calculated with more than 100 platelets. All data are expressed as mean ± SD from 3 experiments. One-way ANOVA with Dunnett’s multiple comparisons test was used to indicate statistically significant differences between groups. *** *p* < 0.001.

Integrin αIIbβ3 is critical for platelet adhesion and spreading [30,31,32]. Therefore, we investigated whether the soy isoflavones affects platelet adhesion and spreading on immobilized fibrinogen. Compared with the control group, the number of platelets adhered to immobilized fibrinogen was not significantly affected by the addition of isoflavones at 100 μM (Figure 4A,B), while 30 μM integrilin reduced the adhering number by 66 % (Figure 4A,B). The average spreading area in genistein and daidzein treated platelets was 63.2 and 63.7 % that of the control group, respectively. These suggest that isoflavones inhibit platelet spreading and lamellipodia formation. The inhibitory effect of genistein and daidzein on platelet spreading was not through GPIb-IX signaling, since both of these compounds inhibited platelet spreading in *GPIbα* deficit platelets (Figure 4C,D).

### 2.5. Genistein and Daidzein Bind to 14-3-3ζ

**Figure 5:**
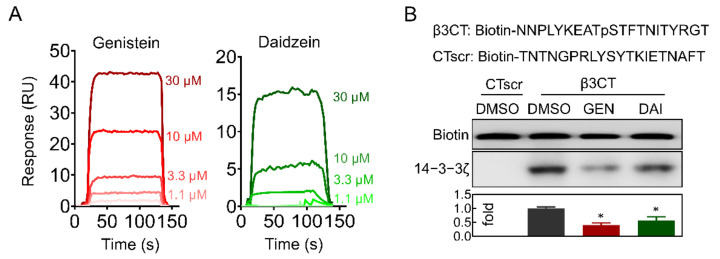
Genistein and daidzein bind to 14-3-3ζ. (**A**) 14-3-3ζ was immobilized on sensor chip CM-5 by amine coupling. Surface plasmon resonance (SPR) analysis of the interaction of genistein (GEN) and daidzein (DAI) with 14-3-3ζ respectively. (**B**) GEN and DAI inhibited 14-3-3ζ binding to integrin β3 cytoplasmic tail. All data are expressed as mean ± SD from 3 experiments. One-way ANOVA with Dunnett’s multiple comparisons test was used to indicate statistically significant differences between groups. * *p* < 0.05.

The activation of integrin outside-in signaling relies on adaptor proteins that bind to integrin cytoplasmic tail, including talin, kindlins, c-Src, Gα13, and 14-3-3ζ [30,31]. Surface plasmon resonance (SPR) measurements indicated that both genistein and daidzein bound to 14-3-3ζ proteins with the estimated equilibrium dissociation constants (KD) of 5.2 × 10^−5^ and 2.1 × 10^−4^ M, respectively (Figure 5A). Binding of recombinant 14-3-3ζ to cytoplasmic domain of integrin β3 (β3CT) was significantly inhibited by these isoflavones at 30 μM (Figure 5B), suggesting the binding of isoflavones functionally blocked the complex formation of 14-3-3ζ with the cytoplasmic domain of integrin β3 in vitro (Figure 5B).

### 2.6. Genistein and Daidzein Inhibit 14-3-3ζ-Integrin β3 Complex Formation and Outside-In Signaling Transduction in Platelet

**Figure 6:**
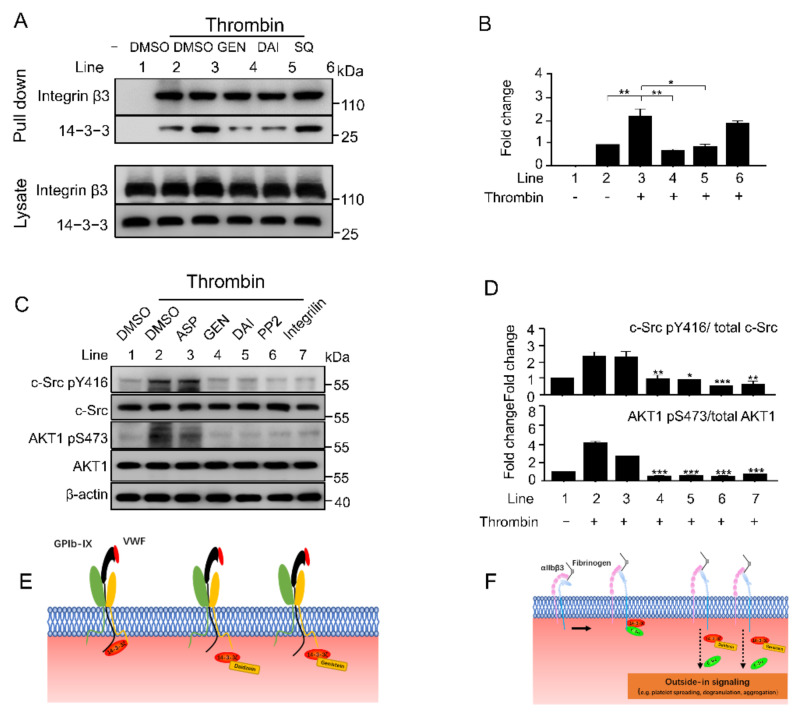
Genistein and daidzein suppress 14-3-3ζ-integrin β3 complex formation and outside-in signaling. (**A**,**B**) Binding of 14-3-3ζ to platelet integrin β3 was inhibited by isoflavones. Platelet pretreated with 0.1% DMSO, 100 μM isoflavones and 3 μM SQ29548 (SQ) for 5 min at 37 °C, then stimulated with 0.04 U/mL thrombin in an aggregometer. Platelets were lysed for co-immunoprecipitation by anti-integrin β3 antibody. (**C**,**D**) Isoflavones suppressed c-Src and AKT1 activation in platelet. Platelets were pretreated with DMSO (0.1 %), aspirin (ASP) and isoflavones at 100 μM, PP2 at 10 μM and integrilin at 3 μM for 5 min at 37 °C, then stimulated with 0.04 U/mL thrombin in an aggregometer. Platelets were lysed after 30 s and subjected to western blot analysis. All data are expressed as mean ± SD from 3 experiments. (**E**,**F**) Genistein and daidzein inhibited GPIb-IX and αIIbβ3 mediated signaling transduction. One-way ANOVA with Dunnett’s multiple comparisons test was used to indicate statistically significant differences between groups. * *p* < 0.05, ** *p* < 0.01, *** *p* < 0.001.

Our previous studies showed that 14-3-3ζ binds to the cytoplasmic domain of integrin β3 and facilitate outside-in signaling transduction [22]. Therefore, we investigated whether genistein and daidzein affect 14-3-3ζ-integrin β3 complex formation in platelet. With the activation of platelets, binding of 14-3-3ζ to integrin β3 was increased by 2.7 times (Figure 6B, line 3). Isoflavones significantly inhibited 14-3-3ζ-integrin β3 complex formation during platelet activation, while the total content of 14-3-3 and integrin β3 in platelet lysate was not changed during the process (Figure 6B). Activation of c-Src is essential for early integrin outside-in signaling and platelet aggregation [30,33]. Isoflavones significantly inhibited the phosphorylation of c-Src Y416 as c-Src inhibitor PP2 did at the early stage of platelet aggregation, while the total c-Src content remained unchanged (Figure 6C,D). Isoflavones also significantly inhibited the activation of AKT pathway (Figure 6C,D), which is considered to be the downstream of platelet integrin outside-in signaling pathway [34]. These results suggested that 14-3-3-integrin β3 complex disrupted by the soy isoflavones affects platelet integrin outside-in signaling pathway.

Based on previous studies, the pharmacokinetics of isoflavones in humans have been exhaustively cognized [35,36,37]. Genistein and daidzein are the most abundant isoflavones in soybeans [38,39,40], which are absorbed through the intestine and liver relative rapidly, and reach the maximum plasma concentration in 2 and 8 h post-intake, and are excreted in urine as glucuronides [41].

Soy isoflavones have been recognized as platelet antagonist and potential antithrombotic, the direct target of these isoflavones in platelet need to be further investigated. In this study, we identified 14-3-3ζ as a novel target of genistein and daidzein. These isoflavones inhibited both GPIb-IX and αIIbβ3 signaling transduction mediated by 14-3-3ζ in platelet (Figure 6E,F).

Early studies have shown that genistein inhibited platelet activation via tyrosine kinase inhibition. However, daidzein, which is not a tyrosine kinase inhibitor, also suppressed platelet aggregation, suggesting the inhibition of tyrosine kinase is not essential for these soy isoflavones mediated platelet inhibition [17,42]. Some flavonoids and their analogues (including genistein) might compete for binding to TxA_2_ receptor, abrogated arachidonic acid and collagen-induced platelet responses [19]. However, in the presence of fibrinogen, isoflavones significantly inhibited high concentration collagen and anti-αIIb TM induced platelet aggregation while TxA_2_ receptor antagonist SQ29548 and adenosine diphosphatase apyrase did not (Figure 3E,F) [22]. This indicates that another target or pathway is involved in genistein and daidzein mediated platelet inhibition.

High concentration of collagen (50 μg/mL) activates integrin αIIbβ3 outside-in signaling independent of PLCγ2, Gαq, TxA_2_ receptor and protein kinase C [43], we found genistein and daidzein significantly inhibited 50 μg/mL collagen-induced platelet aggregation (Figure 3F), suggesting integrin αIIbβ3 outside-in signaling is involved in these soy isoflavones mediated platelet inhibition. The designed peptide anti-αIIb TM directly targets the transmembrane (TM) region of integrin αIIb, turns αIIbβ3 to high affinity state and initiates αIIbβ3-mediated outside-in signaling [29,44]. Anti-αIIb TM induced platelet aggregation was suppressed by genistein and daidzein, but not significantly affected by SQ29548 and adenosine diphosphatase apyrase in the presence of fibrinogen (Figure 3F), indicates αIIbβ3 outside-in signaling was indeed inhibited by these isoflavones. The effect of the soy isoflavones on platelet was not relying on GPIb-IX, as the aggregation and the spreading of the *GPIbα* deficient platelet were also inhibited by these isoflavones (Figure 3G,H and Figure 4C,D).

It has been well documented that 14-3-3ζ is abundant in platelet and plays a critical role in VWF-mediated GPIb-IX signaling transduction [28,44,45]. Our recent study revealed 14-3-3ζ also forms complex with β3 integrin and is very important for integrin outside-in signaling transduction, therefore may be a potential target for antithrombotic treatment without significant bleeding side-effect [22]. Library screening and functionality assay have uncovered some isoflavones or analogues interact with 14-3-3ζ and inhibit platelet aggregation [22], whether genistein and daidzein interact with 14-3-3ζ and inhibit αIIbβ3-mediated outside-in signaling transduction needs further elucidation. SPR and co-immunoprecipitation experiments showed that these soy isoflavones directly bind to 14-3-3ζ (Figure 5A) and functionally inhibited 14-3-3ζ-integrin β3 complex formation both in vitro (Figure 5B) and in vivo (Figure 6A). The interaction of soy isoflavones with 14-3-3ζ blocked both GPIb-IX and αIIbβ3 signaling as the isoflavones inhibited GPIb-IX mediated platelet agglutination (Figure 2) as well as αIIbβ3 mediated platelet aggregation (Figure 3). However, this study could not completely exclude that other proteins or pathways may be also involved in soy isoflavones treated platelets, which may synergistically contribute to the inhibition of platelet.

Soya foods play an important role in typical Asian diets. Early studies have shown that intakes of the isoflavones of soybean and other legumes reach 20–50 mg/d or even higher: 102 mg/d [45]. The concentration of isoflavones is 10-60 μM in adult. This may be one of the reasons why the prevalence of cardiovascular diseases is relatively lower in Asia [46].

## 3. Materials and Methods

All the animal experimental protocols in this work were authorized by the Institutional Animal Care and Use Committees at Kunming Institute of Zoology, Chinese Academy of Sciences (Approval ID: SMKX-20171118-157). C57BL/6 male mice at age of 6–8 weeks (19–25 g) were purchased from the Laboratory Animal Research Center of Kunming Medical University, Kunming, China.

### 3.1. GPIbα Deficient (GPIbα-/-) Mice

The murine *GPIbα* gene is composed of two exons. We replaced the entire *GPIbα* coding sequence (9.0 kb *Hind*III DNA fragment) with a phosphoglycerate kinase-neomycin-resistance (*neo^r^*) cassette (6.7 kb *Hind*III DNA fragment) in murine embryonic stem (ES) cells. Southern analysis of ES cells suggests the successful replacement of the *GPIbα* gene, and Northern analysis indicates the complete absence of *GPIbα* mRNA in homozygous-deficient offspring mice [47,48,49].

### 3.2. Ex Vivo Perfusion Chamber

To study the effect of soy isoflavones on thrombus formation under shear force, we used an ex vivo perfusion chamber system as previously described [50]. Briefly, µ-Slide (µ-Slide VI 0.1, ibidi, Madison, WI, USA) were coated with 100 μg/mL collagen (NC9533954, Thermo Fisher, Waltham, MA, USA) for 2 h at room temperature. Heparinized whole blood was collected from healthy C57BL/6 male mice, pretreated with the soy isoflavones (200 μM) for 10 min at 37 °C and fluorescently labelled by DiOC_6_ (1 μM, Sigma Alderich, Billerica, MA, USA). The platelets were incubated with isoflavone compounds and their solvent (DMSO) for 5 min at 37 °C. Then the blood was perfused over the collagen-coated surface using a syringe pump (Harvard Apparatus, USA) under the shear rate of 300 s^−1^ or 1800 s^−1^ for 5 or 3 min, respectively. Platelet aggregation and thrombus formation were recorded in real-time with Zeiss Axiovert 135-inverted fluorescence microscope (60 X-W objective). Quantitative dynamics of platelet fluorescence intensity were acquired by SlideBook software (Intelligent Imaging Innovations Inc., Denver, CO, USA).

### 3.3. Platelet Preparation and Aggregation

Platelet preparation from wild type (WT) and *GPIbα* deficient (*GPIbα^−/−^*) mice were performed as we previously described [47,51]. Platelet aggregation assay was performed as before [22,52]. Animal protocol for this study was reviewed and approved by the Animal Care and Use Committee at Kunming Institute of Zoology, Chinese Academy of Sciences.

### 3.4. Surface Plasmon Resonance (SPR) Analysis

SPR was performed as previously described [53]. Briefly, recombinant 14-3-3ζ was immobilized on sensor chip CM-5 by amine coupling, genistein and daidzein in HBS-EP+ running buffer was applied to the chip with the flow rate of 10 μL/min and the real-time binding signal was recorded by BIAcore 3000 (GE, Boston, MA, USA). The KD (equilibrium dissociation constant) for binding was calculated using a Langmuir model with the Biacore evaluation software provided by the manufacturer.

### 3.5. Streptavidin–Magnetic Beads Pulldown Assay

The interaction of 14-3-3ζ with the cytoplasmic tail of integrin β3 was carried out as previously described [22]. Briefly, 1 μg biotin-conjugated peptide β3CT (biotin-NNPLYKEATSTFTNITYRGT) was incubated with streptavidin-conjugate magnetic beads (CST, 5947S). Then the beads were incubated with 1 μg recombinant 14-3-3ζ in binding buffer (50 mM Tris, pH 7.4, 150 mM NaCl, 10 mM MgCl_2_, 10 % (*v*/*v*) glycerol) with or without soy isoflavones for 4 h at 4 °C. The protein/peptide was degeneration in 5 × SDS loading buffer (BL502A, Biosharp, Hefei, Anhui, China) and separated by 12 % SDS-PAGE, and further blotted by streptavidin-HRP and 14-3-3ζ antibody.

### 3.6. Immunoprecipitation and Western Blot

Platelets (2 × 10^9^ /mL) pretreated with genistein and daidzein were elicited by 0.04 U/mL thrombin for 30 s, then lysed with ice-cold NP-40 lysis buffer (10 mM Tris, pH 7.4, 150 mM NaCl, 10 mM MgCl_2_, 1 % (*v*/*v*) NP-40, 10 % (*v*/*v*) glycerol) with protease inhibitors (P8340, Sigma) and phosphatase inhibitors (P5726, Sigma Alderich, Billerica, MA, USA). The lysates were degenerated for 5 min at 90 °C in SDS loading buffer, subjected to 10 % SDS-PAGE and blotted with c-Src, c-Src pY416, AKT1 and AKT1 pS473 antibodies. For immunoprecipitation experiment, the lysates were incubated with integrin β3 antibody (2 µg) or isotype-matched control IgG (2 µg) overnight at 4 °C, followed by the addition of protein A conjugated magnetic beads (10002D, Thermo Fisher, Waltham, MA, USA). After 3 times washes with NP-40 lysis buffer, bound proteins on magnetic beads were eluted by 50 mM Glycine (PH 2.8) and degenerated for 10 min at 70 °C in SDS loading buffer for western blot analysis.

### 3.7. Platelet Spreading and Confocal Microscopy

Platelet spreading was measured as we previously described [54]. Briefly, washed platelets were allowed to adhere and spread on fibrinogen (16088, Cayman, Ann Arbor, MI, USA) coated coverslips at 37 °C in the absence or presence of genistein and daidzein. Then platelets were fixed in 4 % paraformaldehyde at room temperature for 15 min, permeabilized in 0.3 % Triton X-100 (T0694, Amresco, Radnor, PA, USA) for 10 min, and stained with FITC labeled phalloidin (40735ES75, Yeasen, Shanghai, China). Platelet numbers and areas were viewed with confocal microscope A1 MP+ (Nikon, Tokyo, Japan) and measured by ImageJ 1.35 h (National Institute of Mental Health, Bethesda, MD, USA) software.

### 3.8. Statistical Analysis

Image J 1.35h software was used to analyze and quantify Western blot bands, platelet spreading area and blood clot retraction area; Flow Jo V10 software (Becton, Dickinson & Company, Ashland, OR, USA) was used to analyze the average fluorescence intensity of platelets bound to FITC-labeled fibrinogen. Statistical significance was assessed by Student’s *t*-test, and GraphPad Prism 6.1 software (GraphPad Software, San Diego, CA, USA) was used for analysis and graphing. The statistical results were expressed as mean ± SD, and it is statistically significant when *p* < 0.05.

## 4. Conclusions

Our study presents evidence that soy isoflavone genistein and daidzein target 14-3-3ζ and inhibit both GPIb-IX and αIIbβ3 signaling transduction mediated by 14-3-3ζ in platelet. Soy isoflavone or isoflavone-rich dietary may potentially contribute to the prophylaxis or treatment of thrombosis or CVD that related with abnormal platelet activation.

## Data Availability

The datasets used and/or analyzed during the current study are available from the corresponding author on reasonable request.

## Sample Availability

Samples of the compounds are available from the authors.
